# Exploring the role of CDCA4 in liver hepatocellular carcinoma using bioinformatics analysis and experiments

**DOI:** 10.1097/MD.0000000000038028

**Published:** 2024-05-03

**Authors:** Changfu Liang, Kaijun Long, Wenhao Zheng, Riqiang Zhong, Zhangrui Li, Shengwei Zhu, Shijing Gu, Chuangshi Zhu, Yan Yang

**Affiliations:** a Department of Hepatobiliary and Pancreatic Surgery, The First Affiliated Hospital of Hainan Medical College, Haikou, China.

**Keywords:** CDCA4, immune microenvironment, liver hepatocellular carcinoma (LIHC), prognosis, TCGA

## Abstract

Liver hepatocellular carcinoma (LIHC) encompasses diverse therapeutic approaches, among which targeted therapy has gained significant prominence in recent years. The identification of numerous targets and the increasing clinical application of targeted drugs have greatly improved LIHC treatment. However, the precise role of CDCA4 (Cell Division Cycle Associated 4), as well as its underlying mechanisms and prognostic implications in LIHC, remains unclear. CDCA4 expression levels in LIHC were analyzed using multiple databases including the cancer genome atlas (TCGA), gene expression profiling interactive analysis (GEPIA), and ULCAN, as well as the datasets E_TABM_36, GSE144269, GSE14520, and GSE54236. The prognostic value of CDCA4 was then evaluated. Subsequently, the association between CDCA4 and immune cells was investigated. Enrichment analysis (GSEA) was utilized to investigate the functional roles and pathways linked to CDCA4. Additionally, the methylation patterns and drug sensitivity of CDCA4 were examined. A predictive model incorporating immune genes related to CDCA4 was developed. The TISCH dataset was used to investigate the single-cell expression patterns of CDCA4. Finally, validation of CDCA4 expression levels was conducted through RT-PCR, Western blotting, and immunohistochemistry. CDCA4 exhibited significant overexpression in LIHC and demonstrated significant correlations with clinical features. High expression of CDCA4 is associated with a poorer prognosis. Analysis of immune infiltration and enrichment revealed its association with the immune microenvironment. Furthermore, its expression is correlated with methylation and mutation patterns. CDCA4 is associated with 19 drugs. Prognostic models utilizing CDCA4 demonstrate favorable effectiveness. T cell subtypes were found to be associated with CDCA4 through single-cell analysis. The conclusive experiment provided evidence of significant upregulation of CDCA4 in LIHC. The high expression of CDCA4 in LIHC is associated with prognostic significance and is highly expressed in T cell subtypes, providing a new therapeutic target and potential therapeutic strategy for LIHC.

## 1. Introduction

The most frequent kind of primary liver cancer, liver hepatocellular carcinoma (LIHC), is also the fourth biggest cause of cancer-related death worldwide. It is characterized by a concealed onset, often diagnosed at an advanced stage when typical clinical symptoms manifest, making treatment challenging and resulting in high mortality rates.^[1,2]^ A number of risk factors, such as exposure to aflatoxins, hepatitis B virus infection, and cirrhosis (chronic liver injury owing to fibrosis), contribute to the development of LIHC.^[3]^ Although “curative surgery” is the preferred approach for early-stage LIHC, approximately 70% of patients encounter tumor recurrence within 5 years post-surgery.^[4]^ Consequently, due to the high recurrence rate post-surgery, LIHC patients have a wide range of treatment options, including liver transplantation, percutaneous ablation, radiotherapy, as well as arterial therapy, systemic therapy, and targeted therapy.^[5]^ In recent years, targeted therapy has significantly improved the treatment of liver cancer, with an increasing number of targets being discovered and targeted drugs being clinically available, offering significant benefits to patients with minimal side effects and favorable treatment outcomes. Exploration of new biomarkers has become imperative, and therefore, research on immune checkpoints and signaling pathways in liver cancer has become a key focus.^[6,7]^

Tumor microenvironment (TME) refers to a process of tumor growth and development that conditions tumor cells to grow, infiltrate, and metastasize. Tumor immune microenvironment (TIME) refers to the presence of many immune cells, surrounding blood vessels, and extracellular matrix based on the TME. Tumors and their surroundings are closely related, and tumors can affect their microenvironment by releasing cell signaling molecules that promote angiogenesis and immune tolerance. Cytotoxic T lymphocytes (CTLs) have been identified as the “main force” in killing tumors in LIHC. Their high level of infiltration indicates a favorable prognosis for patients. B cells play a crucial role in adaptive immunity and dominate humoral immune responses. Studies have shown that B cells mainly distribute in the invasive front region of the TME in liver cancer. Tumor-associated neutrophils (TANs) are highly plastic immune cells. Research has demonstrated a negative correlation between the number of TANs in liver cancer and patient survival time.^[8–12]^

CDCA4 (Cell Division Cycle Associated 4), also referred to as HEPP/SEI-3/TRIP-Br3, is a member of the CDCA gene family. It has been demonstrated that CDCA4 exerts influence over various cellular processes such as cell cycle, proliferation, and apoptosis via the regulation of p53, E2F, and JUN. Additionally, the involvement of CDCA4 in tumor occurrence, development, drug resistance, and prognosis has been observed, with its roles varying across different cancer types. In non-tumor contexts, lower expression levels of CDCA4 have been associated with inhibited myocardial cell proliferation and displayed a negative correlation with heart failure severity in patients.^[13–17]^ CDCA4 has been confirmed to participate in tumorigenesis and metastasis in various cancers. However, its role in primary liver cancer remains uncertain. The expanding realm of genomics and molecular biology research is increasingly dependent on the use of bioinformatics analysis methods. The application of computational strategies for the storage, retrieval, and analysis of biological data has proven instrumental in enhancing our understanding of gene structure, gene expression, gene mutations, genetic features, and target therapeutics. Bioinformatics serves as a critical bridge connecting biological data with meaningful insights. The continued integration of bioinformatics in genetic research will undoubtedly catalyze future discoveries, providing deeper understanding of genetic mechanisms and paving the way for novel therapeutic strategies.^[18–23]^

Investigating the expression of CDCA4 and its prognostic value in liver cancer is the aim of this investigation, along with its influence on the immune microenvironment. Through this investigation, we aim to elucidate the associations between CDCA4 and potential targets or signaling pathways pertinent to the onset and progression of liver cancer.

## 2. Materials and methods

### 2.1. Expression level analysis of CDCA4

We obtained pan-cancer expression matrices and clinical information, including LIHC, from the cancer genome atlas (TCGA) database through the UCSC-XENA database (https://xena.ucsc.edu/). The expression of CDCA4 in various cancers was examined. Validation of the CDCA4 expression levels in LIHC was done using the databases ULCAN (http://www.ualcan.path.uab.edu/) and gene expression profiling interactive analysis (GEPIA) (http://gepia.cancer-pku.cn/). Furthermore, the E_TABM_36 dataset from the ArrayExpress database (https://www.ebi.ac.uk/biostudies/arrayexpress) and the GSE144269, GSE14520, and GSE54236 datasets from the GEO database (https://www.ncbi.nlm.nih.gov/geo/) were employed to further investigate the expression level of CDCA4 in LIHC. Additionally, an investigation of CDCA4 expression levels at various LIHC stages was carried out, and the SolvingLab and UALCAN databases were used for validation.

### 2.2. Prognostic analysis of CDCA4

Univariate Cox regression analysis was carried out to assess the impact of CDCA4 on patient prognosis, specifically in LIHC. The association between CDCA4 and overall survival (OS) was investigated using the Kaplan–Meier method. Additionally, the expression of CDCA4 in LIHC and its prognostic implications were validated utilizing the GEPIA, UALCAN, and PrognoScan databases (http://dna00.bio.kyutech.ac.jp/PrognoScan/).

### 2.3. Impact of CDCA4 on the immune microenvironment

Immune cell scores for 22 different immune cell types in LIHC were determined using the CIBERSORT algorithm. After that, a Spearman correlation analysis was performed to evaluate the connection between immune cells and CDCA4. Furthermore, the R program ESTIMATE was used to calculate the immune score, stromal score, and estimate score for LIHC samples. After that, a Spearman correlation analysis was done to see how these 3 scores and CDCA4 were correlated. Every LIHC sample was divided into high and low expression groups according to the CDCA4 median expression value. We looked at the variations in immune cell expression and scores between the groups with high and low CDCA4 expression. Also investigated was the relationship between CDCA4 and immune cells using the TIMER database (http://timer.cistrome.org/).

### 2.4. Enrichment analysis (GSEA) of CDCA4

The LinKedOmics database (https://www.linkedomics.org/) was employed to analyze genes that displayed significant correlations with CDCA4 in LIHC. The top 50 genes that associated both positively and negatively with CDCA4 were then analyzed using gene ontology (GO) and Kyoto encyclopedia of genes and genomes (KEGG), with the Metascape database being considered. Additionally, GSEA analysis was conducted to investigate the functional and pathway involvement of CDCA4 in GO and KEGG. Furthermore, to further explore the pathways associated with CDCA4, the highly enriched pathways between the high and low CDCA4 expression groups were found using the GSVA technique. Subsequently, a heatmap was generated to visualize these enriched pathways. Correlation analysis was then performed on pathways exhibiting significant differences.

### 2.5. Genomic analysis of CDCA4

The methylation levels of CDCA4 were analyzed using UALCAN. DNA methylation is a chemical change to DNA that can change how genes are expressed without changing the DNA sequence. Gene expression is regulated by its effects on chromatin structure, DNA conformation, DNA stability, and DNA-protein interaction. A methyl group is covalently attached to a cytosine residue 5’ carbon in CpG dinucleotides within the genome during the process of DNA methylation. The association between gene expression and the 3 DNA methyltransferases (DNMT1, DNMT2, DNMT3A, and DNMT3B) was examined using Spearman correlation analysis. DNA repair genes (MMRs) are involved in the cellular mismatch repair mechanism, and loss of key gene function in this process can result in unrepaired DNA replication errors, leading to an increased number of somatic cell mutations. A Spearman correlation analysis was performed to investigate the association between CDCA4 and key MMR genes, specifically MLH1, MSH2, MSH6, PMS2, and EPCAM. Mutation samples from the TCGA database for LIHC were utilized to examine gene mutations. Additionally, chi-square analysis was utilized to evaluate the differences in the frequency of gene mutations between the groups exhibiting high and low CDCA4 expression.

### 2.6. Immunotherapy checkpoint and drug sensitivity analysis of CDCA4

A Spearman correlation analysis was used to investigate the relationship between immunological checkpoints and CDCA4. Data on drug sensitivity and mRNA expression were taken from the CellMiner database. To assess the relationship between drug IC50 values, which are markers of drug sensitivity, and CDCA4 mRNA expression, Spearman correlation analysis was used.

### 2.7. Identification and GSEA of CDCA4-immune related genes

Correlation analysis was performed to identify genes associated with CDCA4 and those linked to immune scores. Venn diagrams were created to determine the overlapping genes between these 2 groups, which represent CDCA4-immune related genes. To investigate the functional annotations and pathways related to these genes, the clusterProfiler R tool was used to perform GO and KEGG analyses.

### 2.8. Construction of the CDCA4-immune related gene risk model

Univariate Cox regression analysis was conducted within the CDCA4-immune related gene set to identify genes correlated with prognosis. Then, we utilized the lasso model to further identify more reliable prognostic-related genes and obtain the key prognostic genes. The samples were divided into high and low-risk groups according to the median risk score using the risk score, which was determined using univariate Cox analysis. The risk model was validated by examining differences in clinical phenotypes.

### 2.9. Relationship between risk score and immune response

The immune infiltration and immune cell scores in the high- and low-risk groups were compared. To forecast cell scores and determine the differences between the high- and low-risk categories, the MCPcounter method was utilized. Additionally, genetic variations associated with immunological checkpoints between the high- and low-risk groups were investigated.

### 2.10. Clinical utility of the risk model

We applied both univariate and multivariate Cox regression, as well as clinical phenotype-specific column plots, to illustrate the clinical utility of the risk model. The practical usefulness of the risk model in forecasting patient outcomes and directing clinical decision-making is demonstrated by these analyses.

### 2.11. Single-cell analysis of CDCA4

The TME is the specific focus of the scRNA-seq database TISCH2 (http://tisch.comp-genomics.org/). It provides detailed annotations of cell kinds down to the individual cell, making TME exploration possible for a wide range of cancer types. We utilized this database to analyze the single-cell expression patterns of CDCA4 in LIHC, providing insights into the cellular landscape and heterogeneity within the TME.

### 2.12. Quantitative real-time PCR

Eight pairs of LIHC and paracarcinoma tissues were obtained from the First Affiliated Hospital of Hainan Medical College. Total RNA was extracted using the RNeasy Mini Kit (QIAGEN, Germany), as per the manufacturer instructions. Reverse transcription was then carried out using the high-capacity cDNA synthesis kit (Takara, China). The mRNA expression levels were measured by qRT-PCR after reverse transcription. Each measurement was performed in biological triplicates, and each experiment was repeated 3 times independently to ensure reproducibility. The primers used for qPCR experiments were as follows: Human CDCA4, forward, 5’-ATTTGAAACGCTGGAGACT-3’; reverse, 5’-CCCATCATGCCTGTCAGTA-3’. Human GAPDH, forward, 5’-TCAAGATCATCAGCAATGCC-3’; reverse, 5’-CGATACCAAAGTTGTCATGGA-3’.Relative gene expression levels were calculated using the 2^ΔΔCt method. Statistical analyses on gene expression data were performed using unpaired Student *t* test. *P* value <.05 were considered statistically significant.

### 2.13. Western blotting analysis

Protein lysates were prepared from the same tissue samples used in the qPCR analysis. Equal volumes of whole tissue lysates were resolved by SDS polyacrylamide gel electrophoresis and transferred onto a PVDF membrane (Pall Corp., Port Washington, NY). This procedure was repeated 3 times for consistency. Following primary antibody incubation on the membranes, the immunoreactive signals were detected with an enhanced chemiluminescence kit (Amersham Biosciences, Uppsala, Sweden) according to the manufacturer guidelines. The relative intensity of the protein bands was quantified using ImageJ software. Statistical analyses on gene expression data were performed using unpaired Student *t* test. *P* value <.05 were considered statistically significant.

### 2.14. Immunohistochemical (IHC) analysis

An IHC assay was conducted to evaluate protein expressions in 8 pairs of LIHC and paracarcinoma tissues. Each IHC staining process was performed independently 3 times. Sections, 4 µm thick, obtained from paraffin-embedded specimens, were baked at 65°C for 30 minutes, followed by deparaffinization with xylene, rehydration, antigen retrieval in EDTA buffer, and sequential incubation with hydrogen peroxide, bovine serum albumin, and primary and secondary antibodies as previously described. The tissue slices were finally submerged in 3-amino-9-ethyl carbazole, dried, counterstained with 10% Mayer hematoxylin solution, and mounted using crystal mount (Electron Microscope Sciences, Hatfield, PA). Semi-quantitative scoring of the intensity of IHC staining was carried out by 2 independent observers blinded to the clinicopathological data. Differences between groups were evaluated using the Student *t* test, with *P* values <.05 considered statistically significant.

## 3. Results

### 3.1. CDCA4 exhibits significant upregulation in LIHC

**Figure 1.**
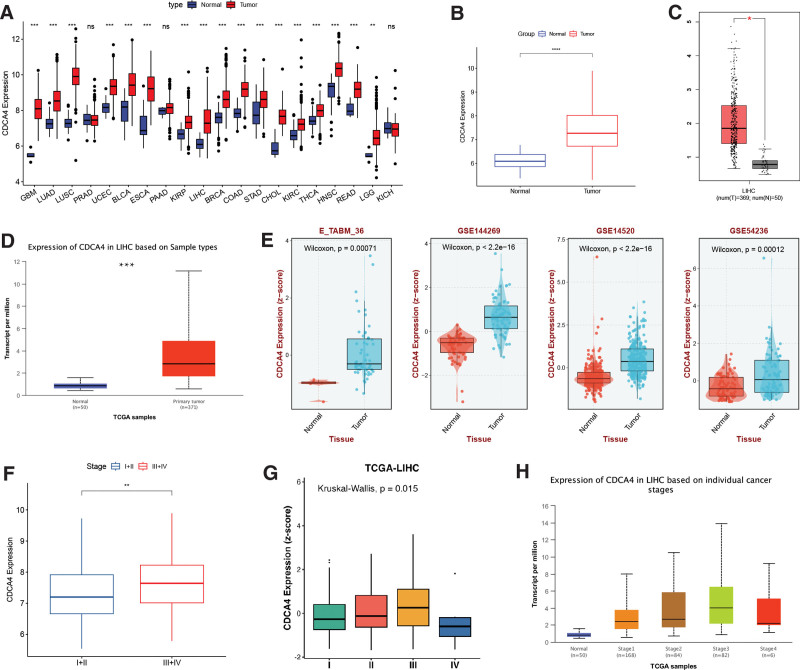
Expression profile of CDCA4. (A) The mRNA expression landscape of CDCA4 in normal and tumor tissue on UCSC-XENA database (the statistical method was Wilcoxon-test. *P < .05, **P < .01, and ***P < .001; ns: no significance). Expression levels of CDCA4 in LIHC from TCGA (B) (the statistical method was Wilcoxon-test. *P < .05, **P < .01, and ***P < .001; ns: no significance), GEPIA (C) (the statistical method was Wilcoxon-test. *P < .05, **P < .01, and ***P < .001; ns: no significance), and UALCAN (D) databases (the statistical method was Wilcoxon-test. *P < .05, **P < .01, and ***P < .001; ns: no significance). Expression levels of CDCA4 in LIHC in the E_TABM_36, GSE144269, GSE14520 and GSE54236 datasets (the statistical method was Wilcoxon-test. A *P* value <.05 was statistically significant) (E). Expression of CDCA4 in different stages of LIHC in TCGA (the statistical method was Wilcoxon-test. *P < .05, **P < .01, and ***P < .001; ns: no significance) (F), SolvingLab (the statistical method was Kruskal-Wallis test. A *P* value <.05 was statistically significant) (G) and UALCAN (the statistical method was Wilcoxon-test. A *P* value <.05 was statistically significant) (H) databases. CDCA4 = cell division cycle associated 4, GEPIA = gene expression profiling interactive analysis, LIHC = liver hepatocellular carcinoma, TCGA = the cancer genome atlas.

CDCA4 demonstrates significant upregulation in 20 different types of tumors, including LIHC (Fig. 1A). Results from TCGA (Fig. 1B), GEPIA (Fig. 1C), UCLAN database (Fig. 1D), and 4 datasets (Fig. 1E) all confirm the significant upregulation of CDCA4 in LIHC. Results from TCGA (Fig. 1F), GEPIA (Fig. 1G), and ULCAN database (Fig. 1H) indicate differential expression of CDCA4 among different stages of LIHC. Table 1 presents the differential expression values of CDCA4 across various clinical stages. This indicates that the expression level of CDCA4 can affect patients with hepatocellular carcinoma, which has further research value.

**Table 1:** The differential expression values of CDCA4 across various clinical stages.

Comparison	Statistical significance
Normal-vs-Stage1	1.62E-12
Normal-vs-Stage2	1.74E-12
Normal-vs-Stage3	9.35E-12
Normal-vs-Stage4	7.35E-02
Stage1-vs-Stage2	9.26E-02
Stage1-vs-Stage3	1.17E-04
Stage1-vs-Stage4	7.70E-01
Stage2-vs-Stage3	6.16E-03
Stage2-vs-Stage4	7.89E-01
Stage3-vs-Stage4	3.24E-01

The statistical method was Wilcoxon-test. CDCA4 = cell division cycle associated 4.

### 3.2. CDCA4 is associated with poor prognosis in LIHC patients

**Figure 2.**
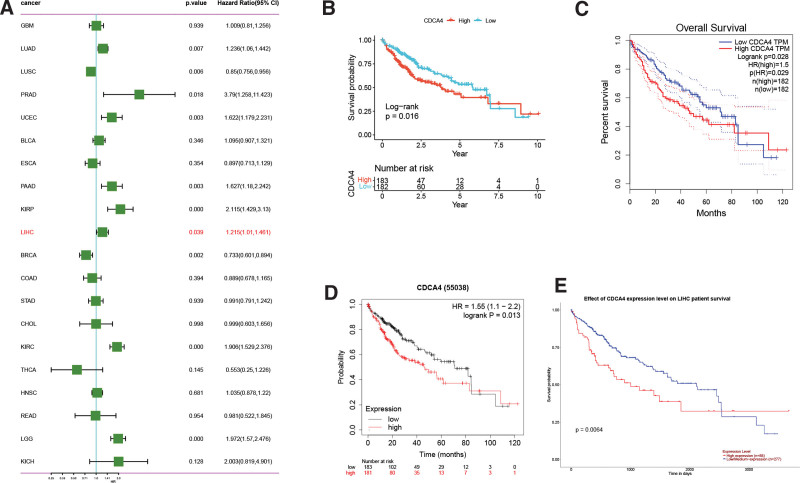
Correlation between CDCA4 expression and prognosis of LIHC patients. (A) Forest plots of hazard ratios of CDCA4 (The statistical method was single factor Cox regression). A *P* value <.05 was statistically significant). Kaplan–Meier survival analysis of CDCA4 expression in LIHC with overall survival (OS) in TCGA (B), GEPIA (C), PrognoScan (D) and UALCAN (E) databases (The statistical method was Kaplan–Meier method. A *P* value <0.05 was statistically significant). CDCA4 = cell division cycle associated 4, GEPIA = gene expression profiling interactive analysis, LIHC = liver hepatocellular carcinoma, TCGA = the cancer genome atlas.

Univariate Cox regression analysis reveals that high CDCA4 expression is correlated with unfavorable prognosis in various cancers, including LIHC (Fig. 2A). KM analyses from TCGA (Fig. 2B), GEPIA (Fig. 2C), PrognoScan (Fig. 2D), and UCLAN (Fig. 2E) databases all provide evidence that high expression of CDCA4 exacerbates adverse prognosis in LIHC patients. This suggests that CDCA4 has potential prognostic value in hepatocellular carcinoma and may be evaluated as an important prognostic indicator.

### 3.3. CDCA4 and its correlation with the immune system

**Figure 3.**
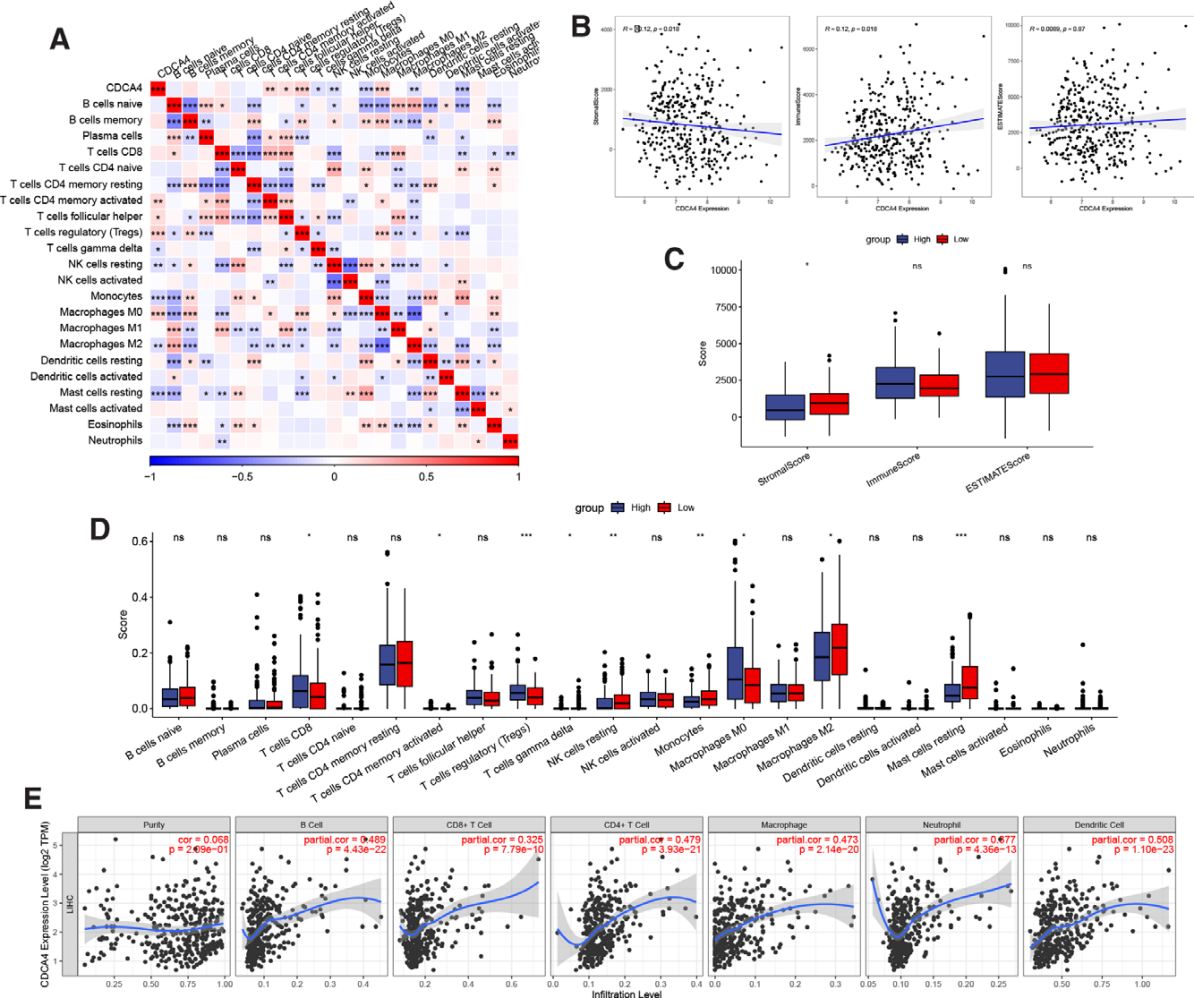
Immune infiltration analysis of CDCA4 in LIHC. (A) Correlation between CDCA4 and LIHC immune cells (Spearman correlation analysis was used. A *P* value <.05 was statistically significant). (B) Correlations between CDCA4 and stromal score, immune score, and estimate score of LIHC (Spearman correlation analysis was used. A *P* value <0.05 was statistically significant). (C) Differences between CDCA4 high and low expression groups in LIHC with stromal score, immune score, and estimate score (the statistical method was Wilcoxon-test. *P < .05, **P < .01, and ***P < .001; ns: no significance). (D)Differences between high and low CDCA4 expression groups in LIHC and immune cell expression (the statistical method was Wilcoxon-test. *P < .05, **P < .01, and ***P < .001; ns: no significance). (E) Correlation between CDCA4 expression level and the level of immune cell infiltration in the TIMER database (Spearman correlation analysis was used. A *P* value <.05 was statistically significant). CDCA4 = cell division cycle associated 4, LIHC = liver hepatocellular carcinoma.

The correlation analysis between CDCA4 and immune cells reveals that CDCA4 is positively associated with Macrophages M0 and negatively associated with Mast cells resting (Fig. 3A). Additionally, CDCA4 shows a significant association with immune score (Fig. 3B). Figure 3C illustrates higher expression of stromal score in the CDCA4 low expression group. Notably, Macrophages M0 exhibit significant differential expression between the high and low CDCA4 expression groups (Fig. 3D). Findings from the TIMER database indicate significant positive correlations between CDCA4 and B cells, CD4 + T cells, CD8 + T cells, Macrophages M0, Neutrophils, and Dendritic cells (Fig. 3E). These results suggest that CDCA4 may play an important role in the immune microenvironment of hepatocellular carcinoma and is closely associated with different types of immune cells.

### 3.4. GSEA of CDCA4

**Figure 4.**
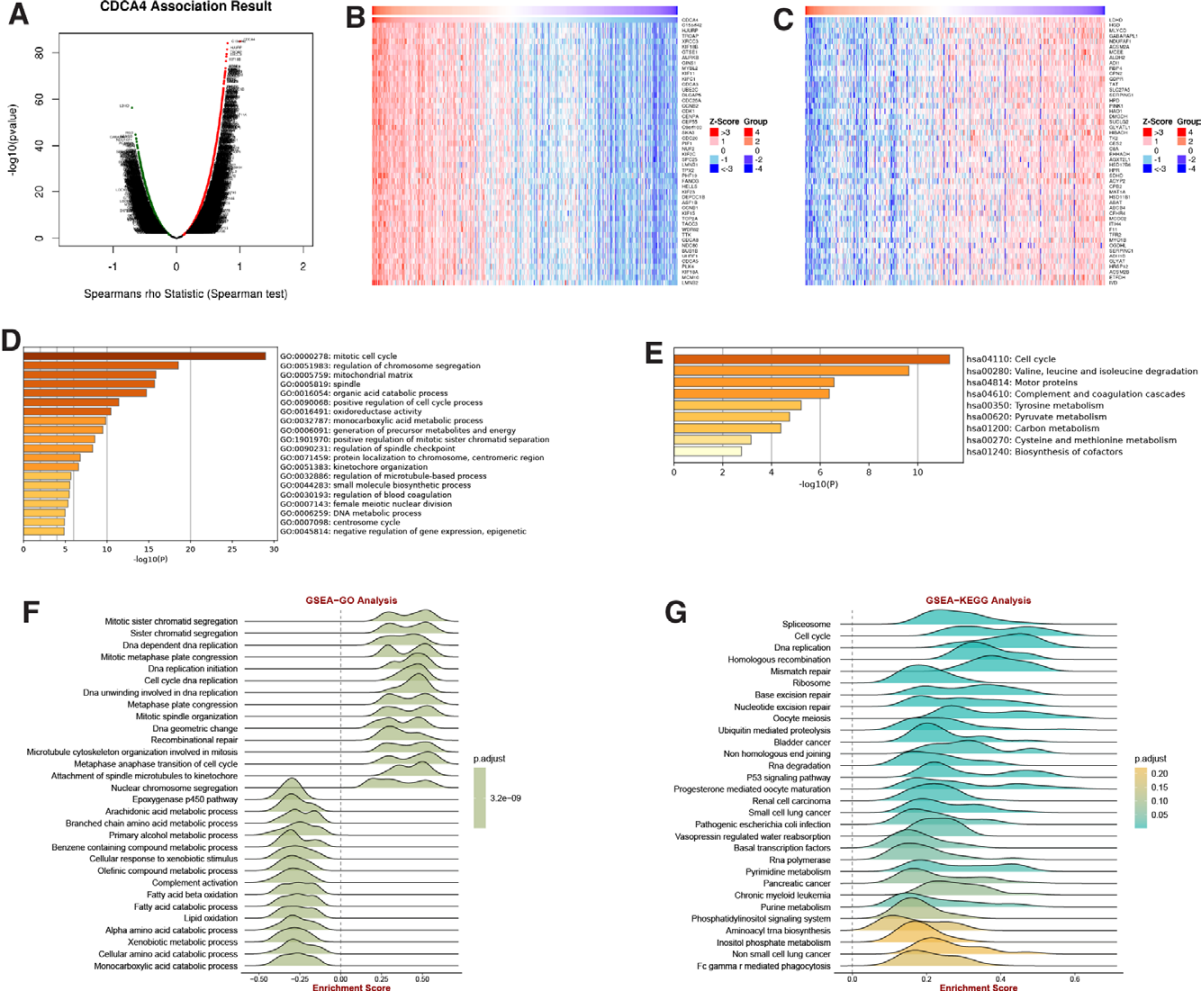
GO, KEGG and GSEA enrichment of DCDA4 in LIHC. (A) Volcano plot of DCDA4 in LIHC. Heatmap of top50 gene expression significantly positively (B) and negatively (C) correlated with CDCA4 in LIHC (Spearman correlation analysis was used. A *P* value <.05 was statistically significant). GO(D) and KEGG(E) enrichment analysis of DCDA4 (The *P* values derived from this test are often corrected for multiple testing, using methods like Bonferroni correction. A *P* value <.05 was statistically significant). GSEA analysis of CDCA4 in GO (F) and KEGG (G) (The statistical method utilized in GSEA is based on the Kolmogorov-Smirnov test. A *P* value <.05 was statistically significant). CDCA4 = cell division cycle associated 4, GO = gene ontology, GSEA = enrichment analysis, LIHC = liver hepatocellular carcinoma.

**Figure 5.**
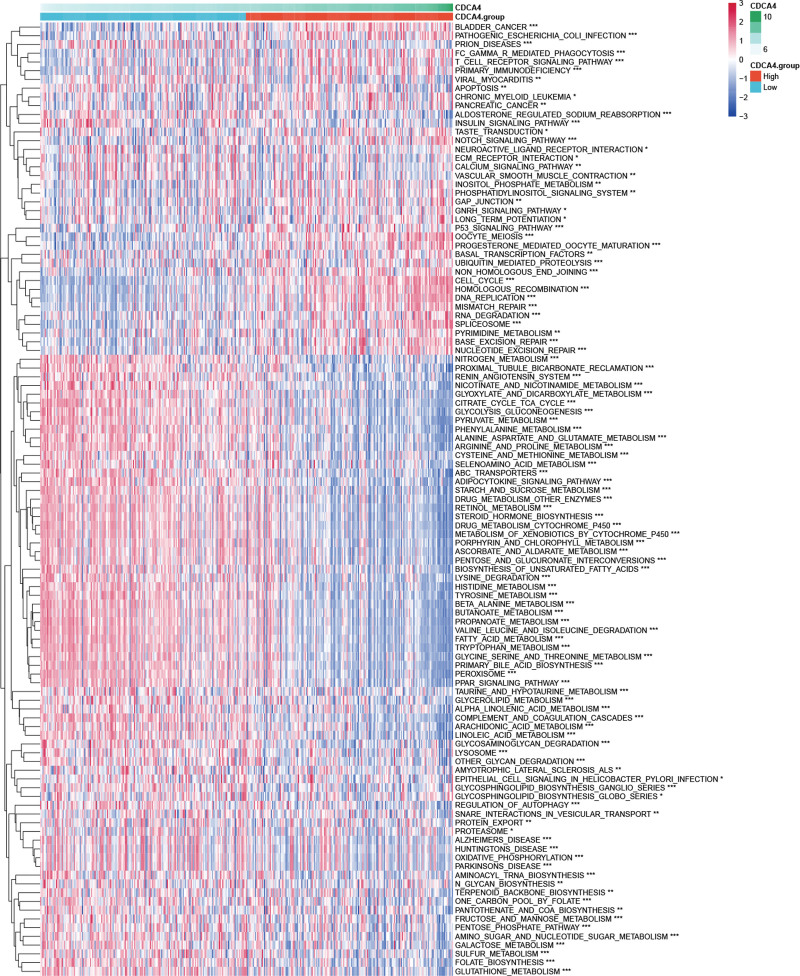
Kyoto encyclopedia of genes and genomes (KEGG) analysis of CDCA4 (the statistical method was t test. *P < .05, **P < .01, and ***P < .001; ns: no significance). CDCA4 = cell division cycle associated 4.

**Figure 6.**
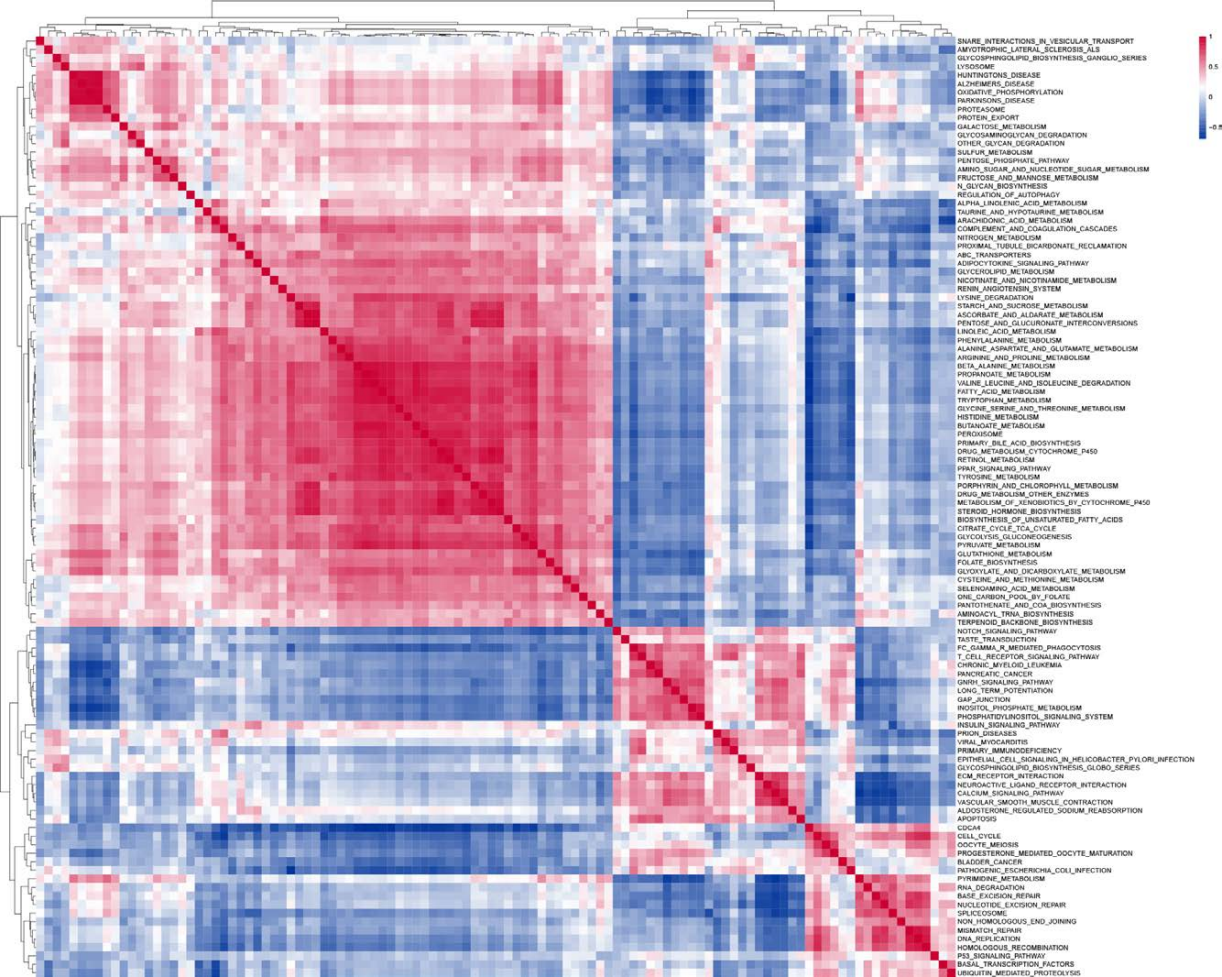
Gene set variation analysis (GSVA) analysis of CDCA4 (Spearman correlation analysis was used). CDCA4 = cell division cycle associated 4.

The volcano plot displays genes significantly associated with CDCA4 in LIHC (Fig. 4A). The heatmap shows the top 50 genes that are significantly positively correlated (Fig. 4B) or negatively correlated (Fig. 4C) with CDCA4 in LIHC. Results from the Metascape database reveal significant enrichment in the mitotic cell cycle pathway (Fig. 4D) for these genes in terms of GO analysis. In terms of KEGG analysis, these genes exhibit significant enrichment in the cell cycle pathway (Fig. 4E). GSEA results demonstrate that CDCA4 significantly activates the metaphase anaphase transition of the cell cycle and inhibits the epaxygenase P450 pathway (Fig. 4F). In the KEGG category, CDCA4 is significantly enriched in non-small cell lung cancer (Fig. 4G). According to GSVA analysis, we found significant associations between CDCA4 and pathways such as Cell cycle, T cell receptor signaling pathway, DNA replication, RNA degradation, and homologous recombination (Fig. 5). Furthermore, there are correlations among these pathways (Fig. 6). These results reveal that CDCA4 may be involved in the regulation of cell cycle and other important biological pathways in hepatocellular carcinoma, further deepening our understanding of CDCA4 function and mechanism of action.

### 3.5. Genomic analysis of CDCA4

**Figure 7.**
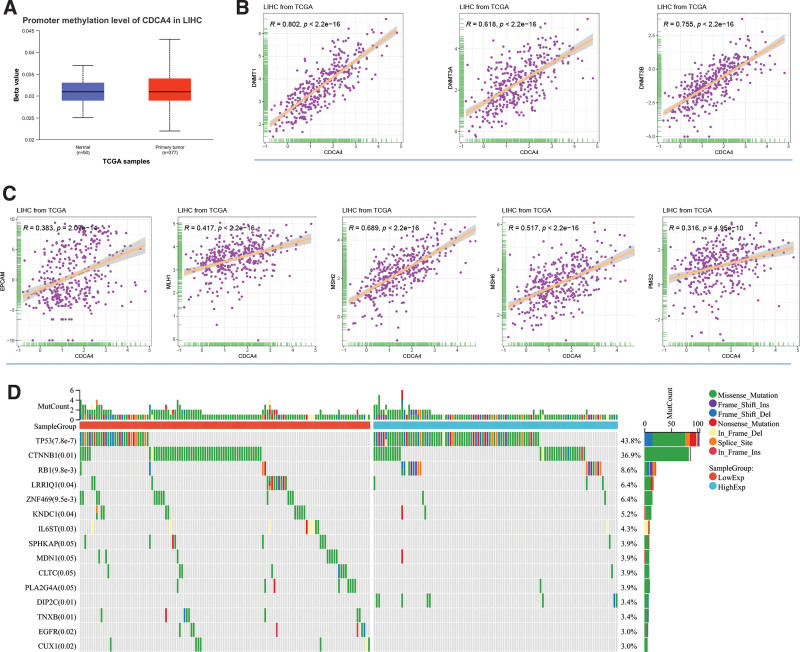
(A) Methylation analysis of CDCA4 in LIHC (the statistical method was Wilcoxon-test. *P < .05, **P < .01, and ***P < .001; ns: no significance). (B) Correlation of CDCA4 with DNA methyltransferases (DNMT1, DNMT2, and DNMT3) (Spearman correlation analysis was used. A *P* value <.05 was statistically significant). (C) Correlation of CDCA4 with MMRs genes (MLH1, MSH2, MSH6, PMS2, and EPCAM) (Spearman correlation analysis was used. A *P* value <.05 was statistically significant). (D) Frequency of mutations of CDCA4 high-low group in LIHC (The statistical method is chi-square test. A *P* value <.05 was statistically significant). CDCA4 = cell division cycle associated 4, LIHC = liver hepatocellular carcinoma, MMRs = DNA repair genes.

CDCA4 shows significant hypomethylation in tumor tissues, which explains the significant upregulation of CDCA4 in LIHC (Fig. 7A). In terms of DNA methyltransferases, CDCA4 is significantly positively correlated with DNMT1 (*R* = 0.802, *P* < 2.2e-16), DNMT3A (*R* = 0.618, *P* < 2.2e-16), and DNMT38 (*R* = 0.755, *P* < 2.2e-16) (Fig. 7B). Regarding MMR genes, CDCA4 exhibits significant positive correlations with EPCAM (*R* = 0.383, *P* < 2.07e-14), MLH1 (*R* = 0.417, *P* < 2.2e-16), MSH2 (*R* = 0.689, *P* < 2.2e-16), MSH5 (*R* = 0.517, *P* < 2.2e-16), and PMS2 (*R* = 0.316, *P* < 4.95e-10) (Fig. 7C). In mutation analysis, we found that TP53, CTNNB1, and RB1 are significantly differentially expressed between the high and low CDCA4 expression groups (Fig. 7D). These results reveal that CDCA4 may play an important role in hepatocellular carcinoma through its association with DNA methylation, MMR genes, and certain mutated genes. These findings provide clues for further investigation of the potential mechanisms of CDCA4 in the development and treatment of hepatocellular carcinoma.

### 3.6. Analysis of CDCA4 in immune checkpoints and drug sensitivity

**Figure 8.**
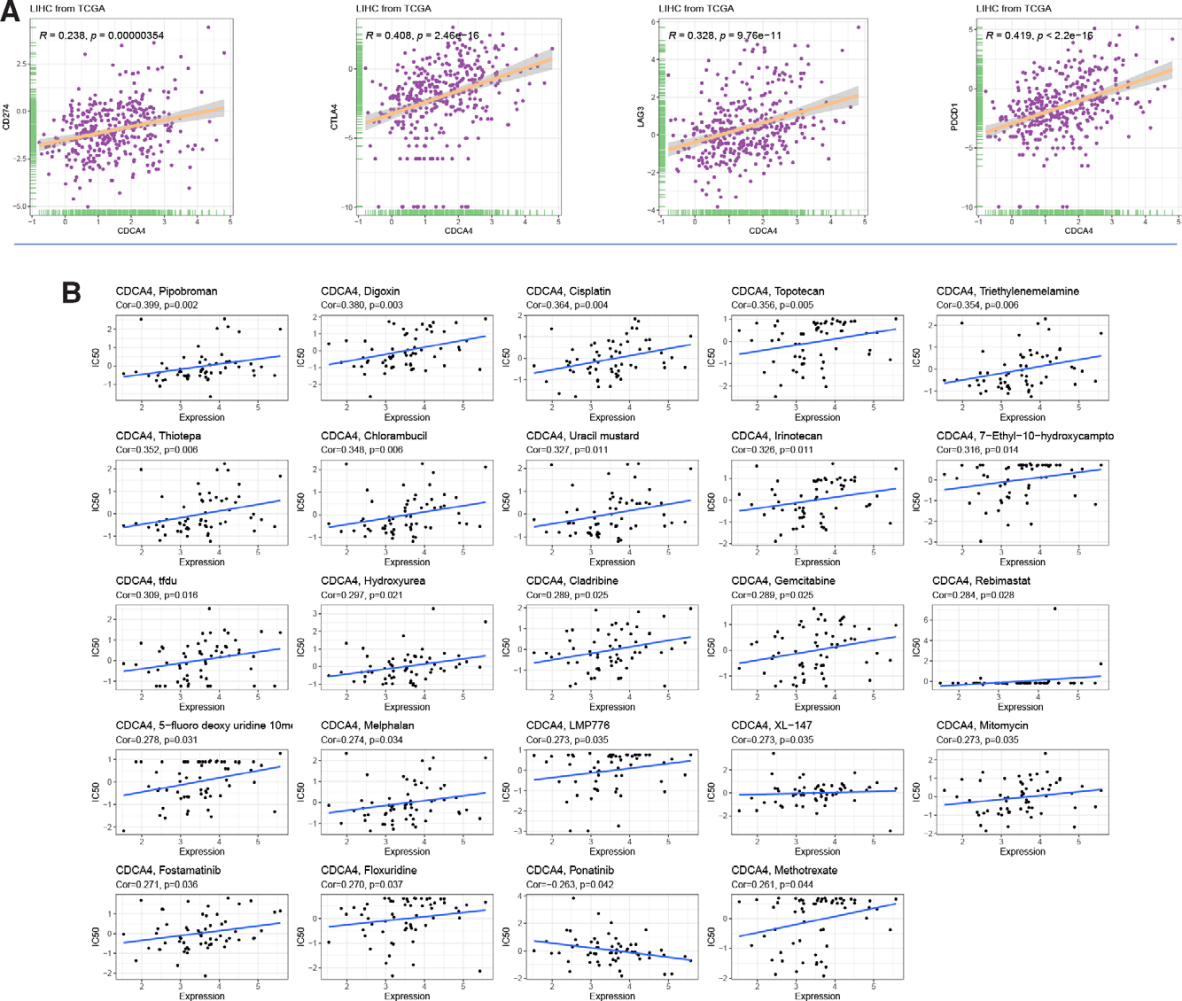
(A) Correlation between CDCA4 and immune checkpoints. (B) Drug sensitivity analysis of CDCA4 in the CellMiner database. (Spearman correlation analysis was used. A *P* value <.05 was statistically significant). CDCA4 = cell division cycle associated 4.

CDCA4 shows significant positive correlations with CD274 (*R* = 0.238, *P* < .00000354), CTLA4 (*R* = 0.408, *P* < 2.46e-16), LAG3 (*R* = 0.328, *P* < 9.76e-11), and PDCD1 (*R* = 0.419, *P* < 2.2e-16) (Fig. 8A). In terms of drug sensitivity, we found 24 genes significantly associated with CDCA4 (Fig. 8B). Digoxin exhibits a significant positive correlation with CDCA4, while AP-26113 shows a significant negative correlation with CDCA4. These results suggest that there may be an association between CDCA4 and the regulation of immune checkpoints and the sensitivity of certain drugs to hepatocellular carcinoma, providing clues for further investigation of the potential role of CDCA4 in the treatment of hepatocellular carcinoma.

### 3.7. Identification and GSEA of CDCA4-immune related genes

**Figure 9.**
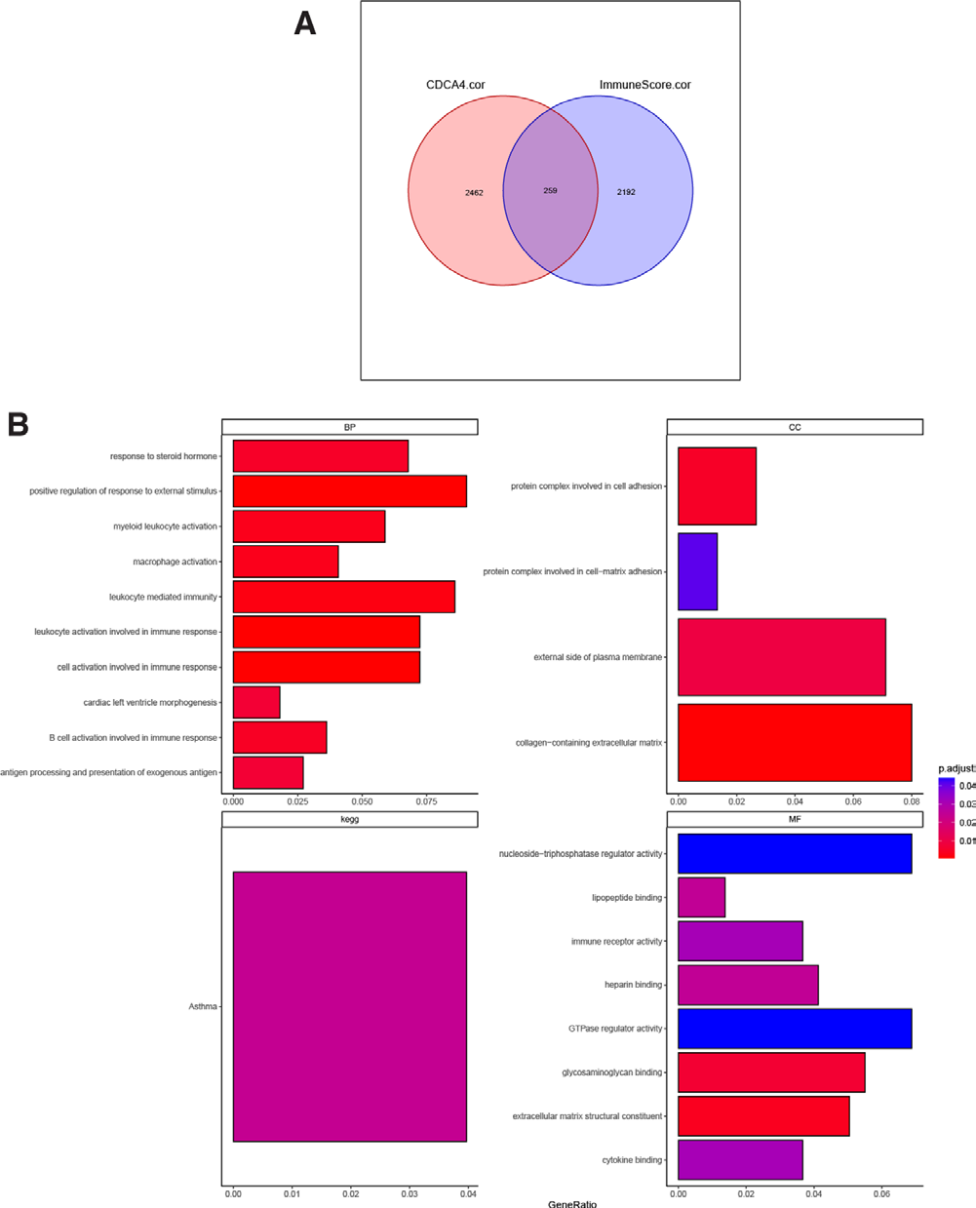
(A) Wayne diagram of common genes (CDCA4 immune related genes) between CDCA4-related genes and immune score-related genes (Spearman correlation analysis was used. A *P* value <.05 was statistically significant). (B) GO and KEGG analysis of CDCA4 immune related genes (The *P* values derived from this test are often corrected for multiple testing, using methods like Bonferroni correction. A *P* value <.05 was statistically significant). CDCA4 = cell division cycle associated 4, KEGG = Kyoto encyclopedia of genes and genomes.

We identified a total of 2462 CDCA4-related genes and 2192 immune score-related genes. Among them, there were 259 genes that were common to both groups, representing the CDCA4-immune related genes (Fig. 9A). These CDCA4-immune related genes showed significant enrichment in various biological processes such as response to steroid hormone, positive regulation of response to external stimulus, and myeloid leukocyte activation (Fig. 9B). In terms of cellular components, these genes were significantly enriched in collagen-containing extracellular matrix, protein complex involved in cell adhesion, and external side of Plasma membrane (Fig. 9B). Additionally, in molecular function, the CDCA4-immune related genes exhibited significant enrichment in GTPase regulator activity, nucleoside-triphosphatase regulator activity, and lipopeptide binding (Fig. 9B). In terms of pathway enrichment (KEGG), the CDCA4-immune related genes were significantly enriched in Asthma (Fig. 9B).

### 3.8. Construction of the risk model

**Figure 10.**
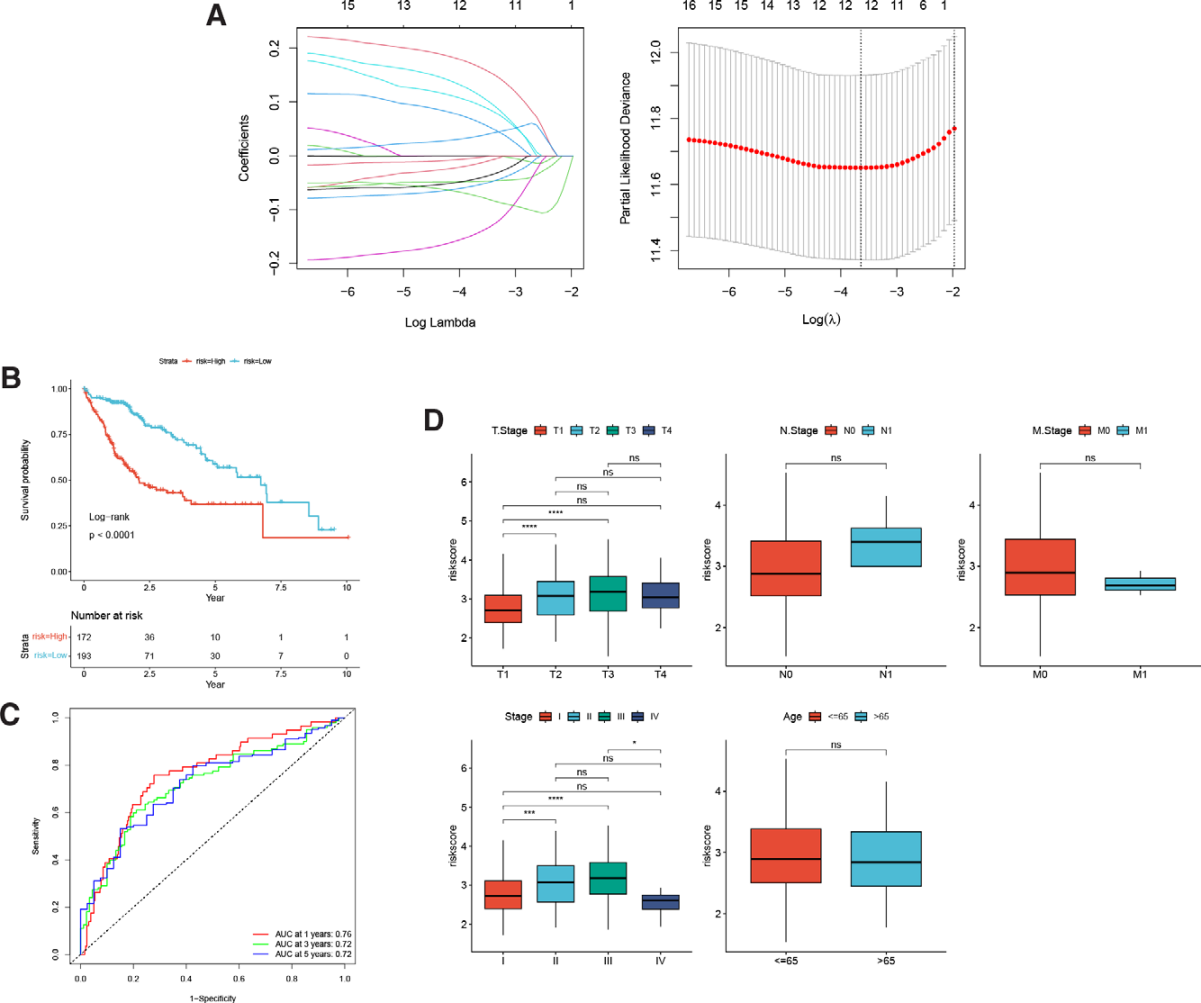
(A) Lasso model. Impact of high and low-risk groups on (B) prognosis, (C) diagnostic efficacy and (D) clinical characteristics (The statistical method was Wilcoxon-test. *P < .05, **P < .01, and ***P < .001; ns: no significance). GO = gene ontology.

In our study, we identified 12 key genes (Fig. 10A) that form a risk model. This risk model is associated with adverse prognosis in patients, indicating a link between the high-risk group and poor outcomes (Fig. 10B). The AUC values for predicting survival rates at 1-year, 3-year, and 5-year time points were found to be 0.76, 0.72, and 0.72, respectively (Fig. 10C). Furthermore, the risk model demonstrates an association with T staging and clinical stage (Fig. 10D). These results suggest that the risk model may serve as a potential prognostic assessment tool to help guide treatment decisions in patients with hepatocellular carcinoma.

### 3.9. Association of the risk model with the immune system

**Figure 11.**
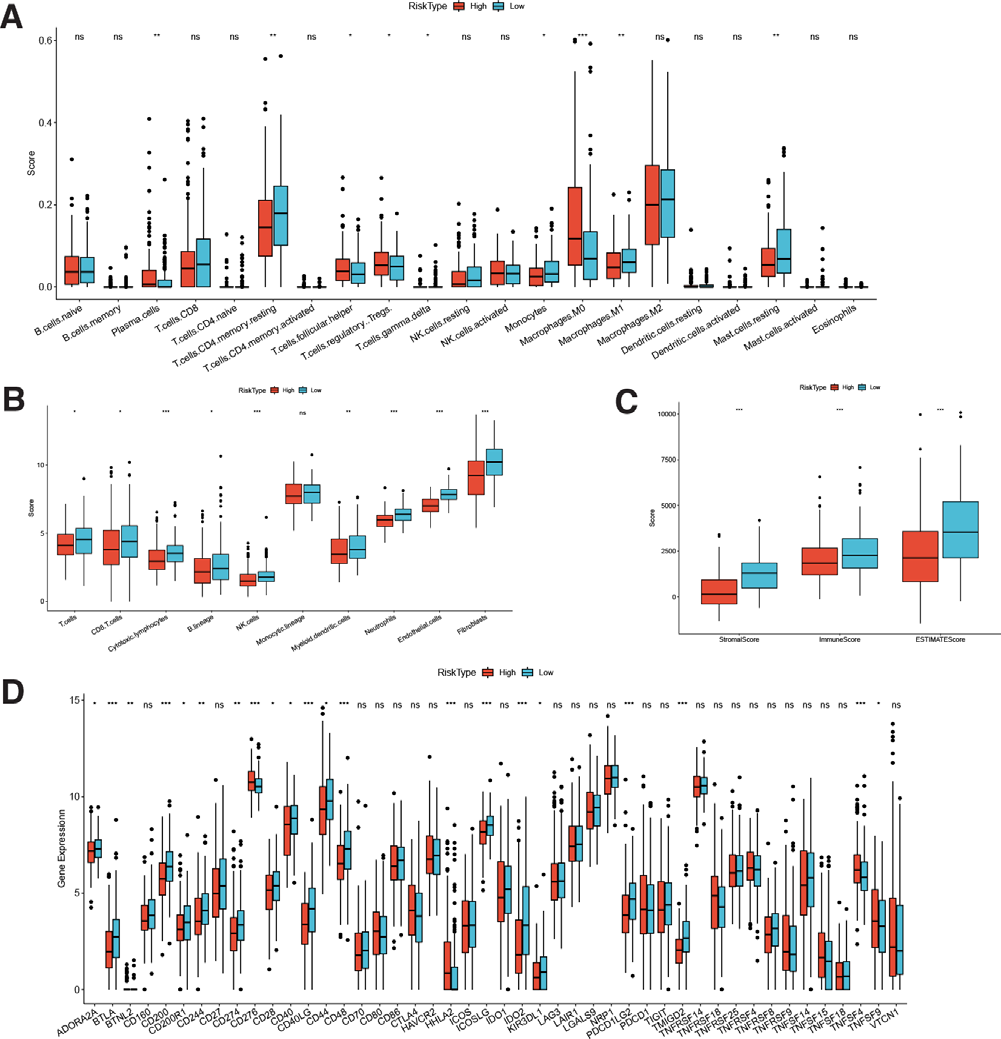
(A) 22 immune cells, (B) 10 immune cells, (C) scores, and (D) immune related genes were differentially expressed at levels in the high- and low-risk groups (The statistical method was Wilcoxon-test. *P < .05, **P < .01, and ***P < .001; ns: no significance).

Figure 11A demonstrates that several immune cell types, including Plasma cells, T cells CD4 memory resting, T cells follicular helper, T cells regulatory Tregs, T cells gamma delta, Monocytes, Macrophages M0, Macrophages M1, and Mast cells resting, show significant expression in the high-risk group. Similarly, Figure 11B indicates that all immune cells, except for Monocytic lineage, exhibit significant expression in the high-risk group. Additionally, in the high-risk group, there are 3 immune scores that show significant expression (Fig. 11C). Furthermore, genes like ADORA2A, BTLA, BTNL2, etc, exhibit significant differential expression in both the high and low-risk groups (Fig. 11D). These results suggest that in the high-risk group, there are differences related to immune cell types, immune scores, and specific gene expression. This further supports the association of CDCA4 with the immune microenvironment in hepatocellular carcinoma and provides more insight into its potential role in immune regulation.

### 3.10. Clinical value of the risk model

**Figure 12.**
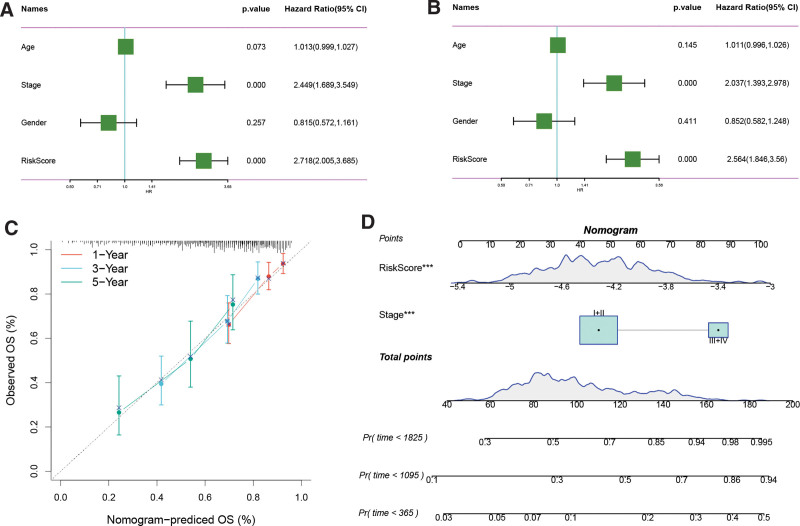
(A) One-factor Cox regression analysis and (B) multifactor Cox regression analysis. calibration curve. (C) calibration curve, and (D) Nomogram of high- and low-risk groups.

Both univariate Cox analysis (Fig. 12A) and multivariate Cox analysis (Fig. 12B) demonstrate that the risk score is associated with adverse prognosis in patients. The nomogram further confirms its clinical predictive value (Fig. 12C–D). These results suggest that risk scores can be used as an important prognostic indicator to assess poor outcomes in patients with hepatocellular carcinoma.

### 3.11. Single-cell analysis

**Figure 13.**
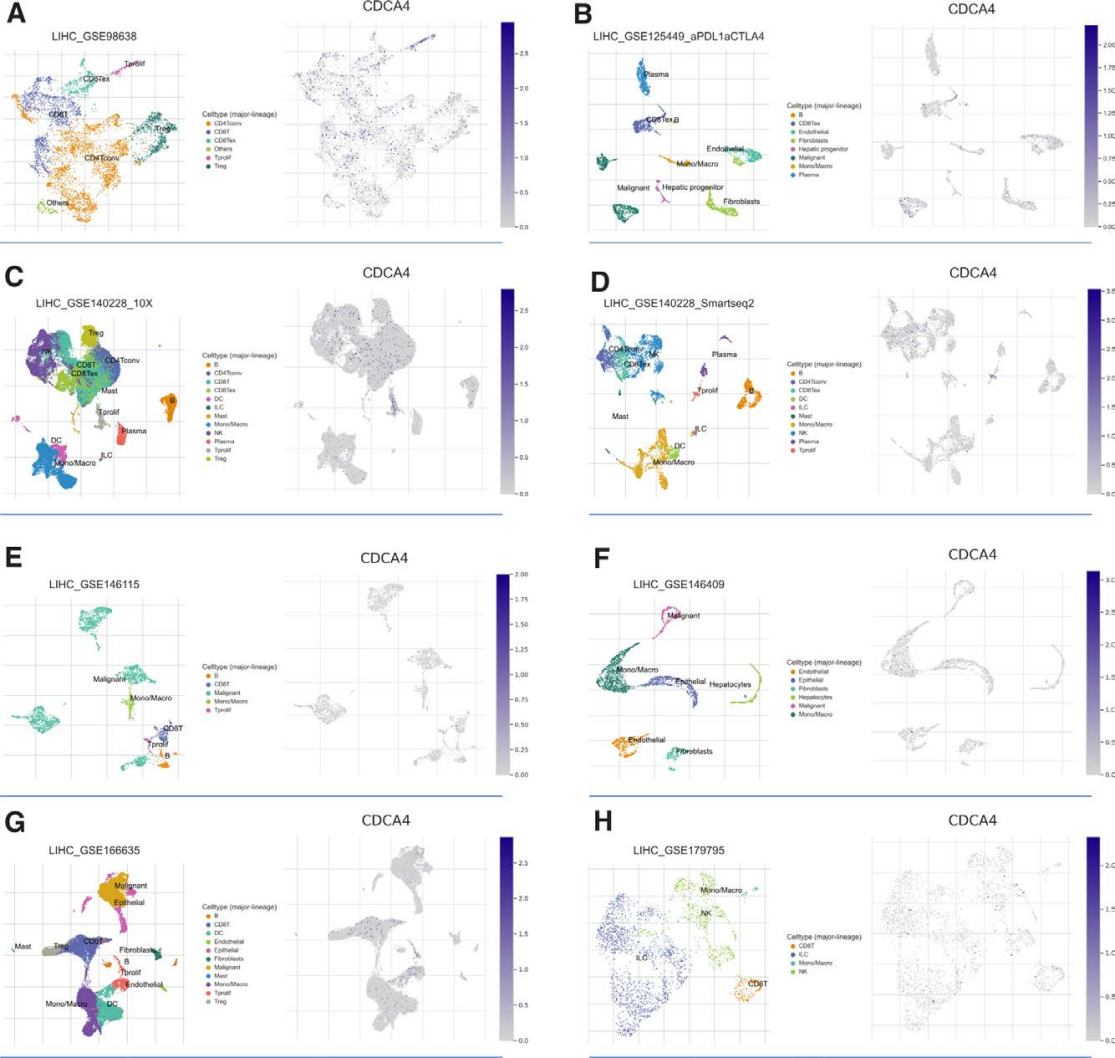
CDCA4 was analyzed at the level of single-cell expression patterns in the (A) GSE98638, (B) GSE125449, (C) GSE140228_10X, (D) GSE140228_Smartseq2, (E) GSE146115, (F) GSE146409, (G) GSE166635, and (H) GSE179795 datasets. CDCA4 = cell division cycle associated 4.

Using 8 single-cell datasets, we investigated the expression patterns of CDCA4 at the single-cell level. In the GSE98638 dataset, our analysis revealed that CDCA4 is primarily expressed in CD8 + T cells and Tprolif cells (Fig. 13A). In the GSE125449 dataset, we observed that CDCA4 is predominantly expressed in CD8Tex cells (Fig. 13B). In the GSE140228_10X dataset, CDCA4 appears to be primarily localized in Tprolif cells (Fig. 13C). In the GSE140228_Smartseq2 dataset, CDCA4 is mainly detected in Tprolif cells and CD8 Tex cells (Fig. 13D). In the GSE146115 dataset, CDCA4 is prominently expressed in CD8 + T cell (Fig. 13E). In the GSE146409 dataset, CDCA4 shows a predominant presence in Mano/Macro cells (Fig. 13F). In the GSE166635 dataset, CDCA4 exhibits significant expression in Treg, CD8 + T and Tprolif cells (Fig. 13G). In the GSE179795 dataset, CDCA4 is predominantly found in CD8 + T cells (Fig. 13H). These results indicate that CDCA4 is highly expressed in T cell subtypes, further supporting its potential role related to immune regulation.

### 3.12. Experimental validation analysis

**Figure 14.**
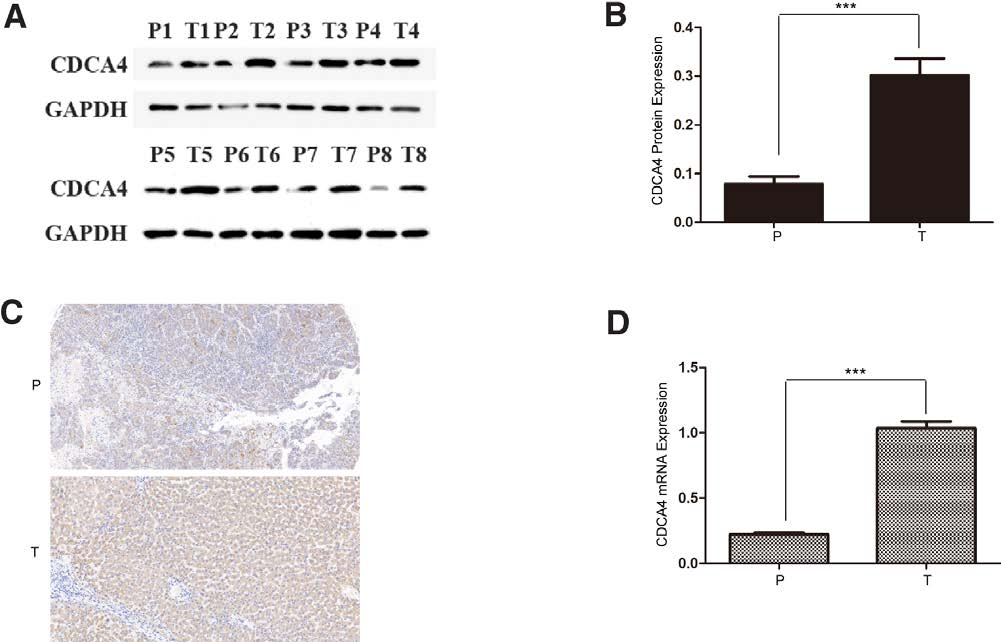
(D) RT-PCR, (A-B) WB and (C) immunohistochemical analyses showed that CDCA4 was significantly highly expressed in LIHC. P: paracancerous; T: tumor. (****P < .001). CDCA4 = cell division cycle associated 4, LIHC = liver hepatocellular carcinoma.

**Figure 15.**
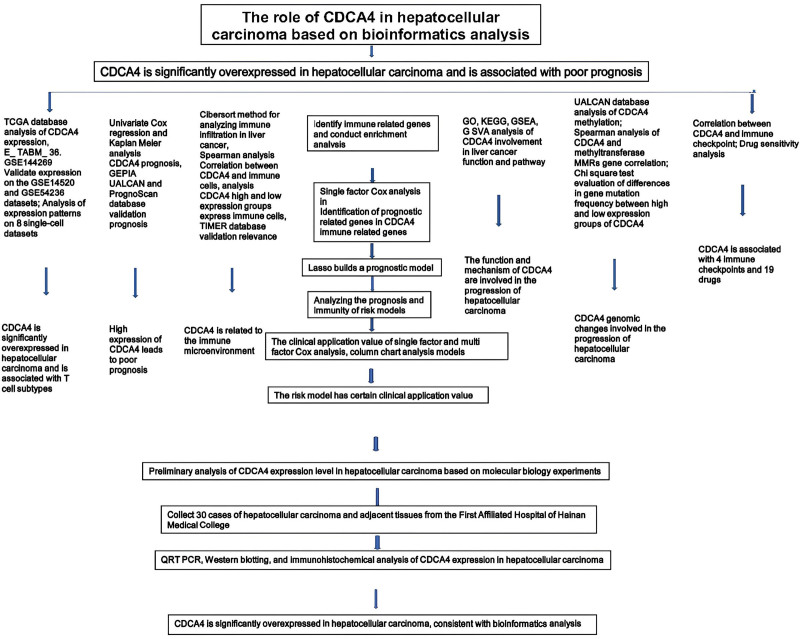
Study flow chart.

Using a combination of techniques including RT-PCR (Fig. 14A), WB (Fig. 14B–C), and immunohistochemical analysis (Fig. 14D), we observed significant overexpression of CDCA4 in LIHC. This is consistent with the results of bioinformatics analysis. The whole research process is shown in Figure 15.

## 4. Discussion

Considerable advancements have been made in the clinical studies for the treatment of LIHC in recent times. Curative therapy including radiofrequency ablation and surgical excision have become the main techniques for treating early-stage LIHC. In contrast, for intermediate and advanced stages of LIHC, a multimodal approach combining local and systemic treatments has been adopted. Notably, a subset of LIHC patients who undergo combination therapies may even achieve conversion surgery, resulting in long-term survival.^[5,7]^ Hence, a comprehensive multidisciplinary treatment approach has proven to be an effective strategy in extending patient survival and enhancing their overall quality of life.^[24]^

Through database screening and experimental validation, it has been demonstrated that CDCA4 is significantly upregulated in LIHC. High expression of CDCA4 is frequently linked to poor prognosis and advanced tumor stage in liver cancer. In this context, CDCA4 is considered an independent prognostic factor in liver cancer, with patients exhibiting lower CDCA4 expression showing better prognosis compared to those with higher expression. Different roles for CDCA4 are played by different forms of cancer. In cases of breast cancer, it lowers apoptosis and encourages the growth of cancer cells. In addition, CDCA4 has been found in cervical cancer to stimulate cell proliferation and act as a tumor promoting gene in cervical cancer.^[13]^ In non-small cell lung cancer, it exhibits tumor-inhibitory effects by regulating the autophagy pathway to inhibit tumor cell migration and invasion.^[17]^ The proliferation and invasion of malignant melanoma cells are also facilitated by CDCA4.^[16]^ Furthermore, lung adenocarcinoma cells are more able to proliferate, migrate, and invade when CDCA4 expression is high.^[14,15]^ Given these diverse roles, CDCA4 shows promise as an important therapeutic target for malignant tumors in the future.

Cancer is a genomic disease characterized by the accumulation of genetic mutations and structural alterations in the genome, leading to the generation of tumor antigens. These antigens can be recognized by the immune system as non-self antigens, triggering a cellular immune response.^[25]^ The immune system, consisting of both adaptive and innate immune cells, infiltrates the TME and plays a vital role in immune surveillance and tumor progression regulation. An effective immune response can destroy malignant cells or impair their function and phenotype. However, cancer cells have developed various mechanisms to evade immune surveillance. This leads to impaired effector function of immune cells and failure of anti-tumor immune responses.^[26]^ Immunotherapy aims to enhance the natural defense mechanisms against cancer cells, and although clinical responses can be sustained in different cancer types, response rates are limited and the underlying mechanisms remain unclear. Immune cells play a fundamental role in immunotherapy, highlighting the importance of understanding the immune infiltrates within the TME for improving response rates and developing novel immunotherapeutic strategies.^[26]^ CDCA4 was significantly and positively correlated with various immune cell types. Immune cells exert dual effects - on one hand, they can play an immune surveillance role and inhibit tumor progression (several studies have shown a poor prognosis associated with Macrophage M0 in LIHC); on the other hand, under the influence of the TME, they can promote tumor phenotypes, allow tumor escape, and even support the TME, thereby facilitating tumor progression. Multiple studies have shown that CD8 + T cells, M0 macrophages, TLSs (Tertiary Lymphoid Structures), TH1 cells, TFH cells, B cells, NK cells, and DCs are associated with a favorable prognosis across various cancer types. The TME can sustain tumor growth and influence immune escape and tumor progression. Discovering immune cells highly correlated with CDCA4 can provide new insights for treatment strategies and predicting patient prognosis.^[27–33]^

In LIHC, there is a close relationship between CDCA4 and several immunological signaling pathways. According to GSVA analysis, we have discovered significant correlations between CDCA4 and pathways such as the Notch signaling pathway, cell cycle, T cell receptor signaling pathway, DNA replication, RNA degradation, and homologous recombination. Additionally, there are interconnections among these pathways, suggesting a link between CDCA4 and the occurrence and development of LIHC. This provides potential breakthroughs for immune therapy in liver cancer. Studies have indicated the tight regulation of hepatocyte interaction through Notch signal transduction. The activation of Notch is closely associated with liver cancer development due to its influence on cancer signal transduction in hepatocytes and cancer stem cells. The Notch signaling pathway plays multiple roles and is clinically associated with the occurrence of liver cancer and liver development functions.^[34–38]^ Research also indicates that inhibitors targeting key regulators of the cell cycle, such as cyclin-dependent kinases, have achieved considerable success in tumor treatment. Among them, Cdk4/6, an important target for tumor therapy, has shown significant efficacy in late-stage metastatic breast cancer, particularly for HR + and Her2-subtypes, with the clinical application of inhibitors such as palbociclib, ribociclib, and abemaciclib. The successful translation of Cdk4/6 inhibitors from basic research to clinical use has brought attention to the cell cycle in tumor cells. On one hand, researchers continue to expand the therapeutic scope of Cdk4/6 inhibitors. Currently, various Cdk4/6 inhibitors are entering clinical trial stages for the treatment of different types of cancers, including leukemia, melanoma, and lung cancer. On the other hand, researchers are also exploring new targets associated with the cell cycle, offering promising prospects for future cell cycle-based therapies.^[39–41]^

DNA methylation represents the most critical form of epigenetic modification.^[42]^ CDCA4 demonstrates significant hypomethylation in tumor tissues, providing an explanation for its heightened expression in tumors. Hypomethylation contributes to increased genomic instability, thereby promoting cellular transformation.^[43]^ Previous studies have indicated that tumors, including LIHC, are driven by genetic mutations and are considered hereditary diseases. Various epigenetic modifications, including DNA methylation, miRNA, lncRNA, and histone acetylation, have been established to play crucial roles in tumor initiation and progression.^[44]^ In terms of mutation analysis, it has been found that TP53, CTNNB1, and RB1 genes are significantly expressed in the high CDCA4 expression group, highlighting their importance in studying tumor signaling pathways. Aberrant DNA methylation serves as a key mediator of epigenetic alterations in LIHC. Abnormal gene methylation is considered one of the key mechanisms underlying the development of LIHC. Typically, abnormal DNA methylation in tumor cells results in the inhibition of genes related to cell cycle regulation, DNA repair, and apoptosis.^[43]^ Studies have found high methylation of genes such as p16, p15, and RASSF1A in early-stage LIHC, suggesting the potential of DNA methylation as a biomarker for early LIHC. However, no specific data confirm its diagnostic value in liver cancer. Abnormal hypermethylation of tumor suppressor gene promoter regions in tumors leads to the silencing of tumor suppressor genes, thus losing their inhibitory effect on tumors. This is currently a major focus of research on tumor methylation.^[45]^ In recent years, DNA methylation detection has become a hot topic in clinical research. Significant advancements have been achieved in the investigation of gene promoter methylation, leading to increased attention toward utilizing specific gene methylation as a biomarker for tumors. With the development and advancement of DNA methylation detection techniques, there has been important progress in the early diagnosis of solid tumors, particularly in liver cancer, cervical cancer, gastric cancer, lung cancer, colorectal cancer, and bladder cancer. Although the feasibility of DNA methylation for early diagnosis of LIHC has been demonstrated, further clinical trials are needed to confirm its clinical applicability. Currently, there is no research combining DNA methylation biomarkers with other detection methods. Combining DNA methylation with classical tumor markers and/or imaging techniques may improve the accuracy of early LIHC diagnosis.^[46]^

In terms of drug sensitivity, we have also observed a significant positive correlation between CDCA4 and drugs such as Digoxin, Cisplatin, 5-fluoro deoxy ridine 10me, Triethylenemelamine, and Thiotepa. This finding provides new insights for subsequent drug treatment choices. On the other hand, Studies have demonstrated a significant association between long-term use of Digoxin in women and an increased risk of breast cancer, specifically ER-positive breast cancer. Digoxin has chemical properties similar to estrogen and may exert estrogen-like effects, thereby increasing the incidence of breast cancer.^[47]^ Furthermore, current use of Digoxin has been associated with a heightened risk of colorectal cancer.^[48]^ Medication adherence is a key factor related to cancer incidence, as specific drugs only impact cancer rates when used for the treatment of chronic diseases that require long-term medication. Users of Digoxin have a higher risk of cancer development compared to those who use beta-blockers, thus emphasizing the need for strict monitoring of Digoxin concentration in individuals using it for extended periods.^[49]^ In addition, research has also indicated that Digoxin appears to be effective in cancer treatment. Digoxin, derived from Digitalis extracts, is known for effectively inhibiting sodium-potassium adenosine triphosphatase (Na+/K+ ATPase). Studies have suggested that Digoxin possesses anti-proliferative properties against prostate cancer and breast cancer cells.^[50]^ Digoxin demonstrates potential therapeutic benefits for non-small cell lung cancer in humans by inhibiting the phosphorylation of a protein kinase implicated in tumor cell survival, proliferation, metastasis, and autophagy within the PI3K/Akt pathway signaling. Nevertheless, further proof is required to support these conclusions.^[51]^ However, the evaluation of Digoxin or other naturally derived cardiac glycosides for clinical cancer prevention should first consider safety concerns. Further research is needed to assess their effectiveness.

Despite the systematic analysis conducted on CDCA4, this study inevitably has limitations. Firstly, the precise significance of CDCA4 expression in various cancers and the exact implications of immune regulation in these cancers are not fully understood, as there is limited research and literature available on CDCA4. Secondly, to fully understand the function of CDCA4 in immunological modulation, further direct clinical trials are needed. Thirdly, the specific mechanisms of CDCA4 in liver cancer remain unclear, and future experiments will be conducted to explore its functions and mechanisms in more depth. Lastly, we will continue to conduct additional experimental validations and search for effective therapeutic targets in the signaling pathways significantly associated with CDCA4.

In conclusion, our study demonstrated that high CDCA4 expression was closely associated with immune infiltration and poor prognosis in hepatocellular carcinoma. Meanwhile, single-cell analysis showed that it was highly expressed in T cell subtypes, suggesting that it may serve as a novel therapeutic target. In addition, the predictive model we developed showed better diagnostic performance. In the future, the development of targeted or novel immunotherapies against CDCA4 may reduce the mortality rate of liver cancer patients. Of course more experimental studies are needed in order to determine the exact mechanisms behind cancer occurrence and progression.

## Acknowledgments

We thank the public databases and patients for its assistance in our research.

## Author contributions

**Conceptualization:** Changfu Liang, Yan Yang.

**Data curation:** Changfu Liang, Zhangrui Li, Shijing Gu.

**Formal analysis:** Changfu Liang, Wenhao Zheng, Yan Yang.

**Funding acquisition:** Yan Yang.

**Investigation:** Kaijun Long, Wenhao Zheng, Riqiang Zhong, Zhangrui Li, Shengwei Zhu, Shijing Gu, Chuangshi Zhu.

**Methodology:** Changfu Liang, Shijing Gu.

**Project administration:** Changfu Liang, Riqiang Zhong, Yan Yang.

**Resources:** Shengwei Zhu.

**Software:** Kaijun Long, Riqiang Zhong, Chuangshi Zhu.

**Supervision:** Yan Yang.

**Visualization:** Kaijun Long, Yan Yang.

**Validation:** Wenhao Zheng, Zhangrui Li, Shengwei Zhu, Chuangshi Zhu.

**Writing – original draft:** Changfu Liang.

**Writing – review & editing:** Changfu Liang, Yan Yang.
